# Effect of Perforator Territory Infarction on Functional Outcome in Patients With Large Vessel Occlusion

**DOI:** 10.1161/STROKEAHA.125.051745

**Published:** 2025-09-19

**Authors:** Yasmin Sadigh, Valerie I. Vogels, Pieter Jan van Doormaal, Iris S.C. Verploegh, Dana Pisica, Diederik W.J. Dippel, Clemens M.F. Dirven, Aad van der Lugt, Charles B.L.M. Majoie, Ruben Dammers, Victor Volovici, for the MR CLEAN-NO IV and MR CLEAN-MED Investigators, Yvo Roos

**Affiliations:** 1 Department of Neurosurgery (Y.S., V.I.V., I.S.C.V., D.P., C.M.F.D., R.D., V.V.), Erasmus MC University Medical Center, Rotterdam, the Netherlands.; 2 Department of Radiology and Nuclear Medicine (P.J.D., A.L.), Erasmus MC University Medical Center, Rotterdam, the Netherlands.; 3 Department of Neurology (D.W.J.D.), Erasmus MC University Medical Center, Rotterdam, the Netherlands.; 4 Erasmus MC Stroke Center and Center for Complex Microvascular Surgery (Y.S., R.D., V.V.), Erasmus MC University Medical Center, Rotterdam, the Netherlands.; 5 Department of Radiology and Nuclear Medicine, Amsterdam UMC, University of Amsterdam, the Netherlands (C.B.L.M.M.).

**Keywords:** cerebral infarction, ischemic stroke, magnetic resonance imaging, thalamus, thrombectomy

## Abstract

**BACKGROUND:**

Knowledge of the anatomic distribution of cerebral perforating arteries and the consequences of ischemia in these territories is limited. This study aims to evaluate the effect of perforator territory ischemia on functional outcome in patients with anterior circulation large vessel occlusion.

**METHODS:**

A post hoc analysis of the Multicenter Randomized Clinical Trial of Endovascular Treatment for Acute Ischemic Stroke in the Netherlands, MR CLEAN-MED (Safety and Efficacy of Aspirin, Unfractionated Heparin, Both, or Neither During Endovascular Stroke Treatment) and MR CLEAN-NO IV (Intravenous Treatment Followed by Intra-Arterial Treatment Versus Direct Intra-Arterial Treatment for Acute Ischemic Stroke Caused by a Proximal Intracranial Occlusion) participants, recruited between January 2018 and January 2021 from 20 European stroke centers, was performed. Patients undergoing endovascular thrombectomy for anterior circulation large vessel occlusion with available posttreatment magnetic resonance imaging were included. Patients were assigned to 3 groups based on infarct location identified on ≤24 hours posttreatment magnetic resonance imaging: cortex group, perforator and insular group, and both groups (cortical alongside insular and perforator territory). Multivariable linear and ordinal regression analyses were performed separately with the National Institutes of Health Stroke Scale score at 24 hours as the primary outcome and modified Rankin Scale at 90 days as the secondary outcome, adjusted for baseline scores and prognostic factors.

**RESULTS:**

Out of 1167 patients, a total of 397 were included. The median age was 71 (interquartile range, 62–79), and 204 (51%) were men. Although no significant association was found in multivariable analysis between perforator territory infarctions and 24 hours National Institutes of Health Stroke Scale, patients with perforator territory ischemia were more likely to have a worse 90-days modified Rankin Scale (median modified Rankin Scale, 2 [interquartile range, 1–3]; common odds ratio, 2.94 [95% CI, 1.73–4.98]). Besides infarct locations, Thrombolysis in Cerebral Infarction grade 2B-3 and Heidelberg bleeding classifications 1c and 2 significantly influenced the 24-hour National Institutes of Health Stroke Scale and 90-day modified Rankin Scale outcomes.

**CONCLUSIONS:**

Patients with anterior circulation large vessel occlusion leading to perforator territory infarctions are more likely to have an unfavorable functional outcome at 90 days. Future research should focus on better visualization of perforating arteries and understanding their functional anatomy to prevent perforator territory ischemia and improve patient outcomes.

The cerebral perforating arteries are generally considered end-arteries that branch off from the main trunks of the circle of Willis and vascularize the deep structures of the brain.^1^ These areas, defined as the deep central core of the hemispheres, comprise, coarsely, the basal ganglia, internal capsule, and thalamus. As traditional anatomic knowledge identifies perforating arteries as end-arteries, their occlusion may lead to irreversible ischemia.^1,2^ Previous studies suggest that chronic occlusion of perforator arteries caused by intimal plaques of large parent vessels contributes to cerebral small vessel disease and lacunar strokes.^1,3,4^ Anatomic studies show a large heterogeneity in the distribution of perforator artery groups and their vascular territories, except for the posterior communicating and anterior choroidal arteries.^2^

Despite their clinical relevance, neither direct angiographic techniques nor 7.0 Tesla magnetic resonance imaging (MRI) has the potential to reliably provide a complete, high-resolution image of perforator anatomy and potential collaterals.^1,5^ Techniques, such as lesion-deficit mapping, have been used to relate lesion location to clinical outcomes.^6^ However, these maps are often limited in spatial resolution and anatomic detailing and may not adequately capture subcortical structures supplied by perforating arteries.^7^ As a result, mapping the vascular territories of different perforator groups and investigating the effects of perforator territory stroke remains a challenge. Due to the incomplete mapping of the perforator territories, the effect of perforator occlusion on long-term clinical outcome and the possibility of collateral circulation has insufficiently been sufficiently investigated.

Additionally, data on the involvement of perforator artery occlusion in studies including patients suffering from acute large vessel occlusion (LVO) is limited. Patients with LVO are more likely to present with both cortical and subcortical ischemia, yet the specific contribution of perforator territory infarction in this population is rarely characterized. To evaluate treatment outcomes, 2 clinically validated measures are widely used in stroke research: the modified Rankin Scale (mRS)^8^ and the National Institutes of Health Stroke Scale (NIHSS).^9^ The mRS captures global disability and is the standard score for assessing functional recovery after stroke, whereas the NIHSS is widely used to quantify stroke severity and neurological deficit in the acute phase. Both scores have demonstrated prognostic value and are essential for assessing the impact of lesion characteristics on recovery.^9,10^ Previous evidence indicates that such perforator territory ischemia may be associated with poor functional outcomes, reflected by worse mRS scores at follow-up.^11^ This study aims to assess the anatomic distribution of perforator territory ischemia and its effect on clinical symptoms and functional outcome in patients with anterior circulation LVO.

## Methods

### Study Design

A retrospective subgroup analysis of data collected by the CONTRAST Consortium (The Collaboration for New Treatments of Acute Stroke) in 2 multicenter clinical randomized controlled trials in the Netherlands—MR CLEAN-MED (Safety and Efficacy of Aspirin, Unfractionated Heparin, Both, or Neither During Endovascular Stroke Treatment) and MR CLEAN-NO IV (Intravenous Treatment Followed by Intra-Arterial Treatment Versus Direct Intra-Arterial Treatment for Acute Ischemic Stroke Caused by a Proximal Intracranial Occlusion)—was performed. MR CLEAN-MED assessed the effects of unfractionated heparin, or platelet inhibitors, against no antithrombotics, and MR CLEAN-NO IV assessed the effect of intravenous r-tPA (recombinant tissue-type plasminogen activator) against no r-tPA in patients who underwent endovascular thrombectomy within 6 hours from onset for a confirmed anterior circulation occlusion.^12,13^ Patients were recruited consecutively from 20 centers in Europe between January 2018 and January 2021. This study was conducted in accordance with the Strengthening the Reporting of Observational Studies in Epidemiology guidelines. The data that support the findings of this study are available from the corresponding author on reasonable request.

### Eligibility Criteria

All patients with computed tomography (CT) angiography confirmed anterior circulation LVO, and available follow-up MRI were included in this study. Patients with suboptimal digital subtraction angiography (DSA) or MRI-image quality due to movement artifacts, no LVO, distal vessel occlusion, or preexisting neurological disease on MRI were excluded, as well as patients who had CT for follow-up.

### Data Collection

Sociodemographic data were collected, including sex, age, history of ischemic stroke, hypertension, diabetes, smoking status, use of antiplatelet agents, vitamin K antagonists, and direct oral anticoagulants. Clinical data included baseline mRS and baseline NIHSS. Treatment variables included administration of intravenous thrombolysis. Reperfusion outcomes were graded according to the Thrombolysis in Cerebral Infarction scale. Time metrics included median time from symptom onset to reperfusion and from symptom onset to hospital arrival. Hemorrhagic complications were categorized using the Heidelberg Bleeding Classification.^14^

### Neurological Deficit and Functional Outcome Measures

The primary outcome was NIHSS full scale at 24 hours of follow-up for assessment of acute neurological status, adjusted for baseline NIHSS. The secondary outcome was the degree of disability on the mRS at 90 days. As described in MR CLEAN-MED and MR CLEAN-NO IV, 90-day mRS data were collected centrally using structured telephone interviews, with legal representatives interviewed when patients were unable to communicate.^12,13^

### Anatomic Outcome Measures

#### Identification of Perforating Artery Involvement

DSA-image sequences were used to identify the occlusion level and involvement of the perforator groups. The occlusion levels were divided in the internal carotid artery (ICA) from the cervical segment to the branching of the posterior communicating, ICA terminus (ICA-t), proximal and distal M1 segment of the middle cerebral artery, M2 superior, and M2 inferior trunk.

#### Infarction Region Assessment

The primary anatomic end point was the infarction location on MRI obtained at 24 hours of follow-up, identified based on diffusion-weighted image and T2-weighted/FLAIR MRI sequences. Three separate regions were defined on MRI: (1) the perforator territory^2^; (2) the insular region, including the external capsule, claustrum, extreme capsule, and insula; and (3) the cortex.

Patients were assigned to 3 groups based on the infarct location identified on MRI and the prognostic significance of these locations: cortex group (only cortical infarctions), perforator and insular group (perforator and insular territory), and both group (cortical alongside insular and perforator territory).

Infarction location assessment was performed by 2 independent researchers (Y.S. and V.I.V.), and all results were cross-checked. Disagreements were discussed in weekly meetings with the senior author (V.V.). Interrater reliability between the 2 independent researchers (Y.S. and V.I.V.) in determining infarction location assessment was measured using Cohen κ. For internal quality assurance, 15% of the DSA and MRI scans were randomly sampled and evaluated by an independent third observer (P.J.D., neuro-radiologist). Agreement percentages between primary observers (Y.S. and V.I.V.) and the third expert observer were calculated.

### Statistical Analysis

#### Descriptive Analysis and Main Outcomes

Absolute and relative frequencies were reported for all categorical variables. Medians and interquartile range (IQR) were calculated for continuous variables. Differences between patients by infarction location (cortex group, perforator and insular group, and both groups) were tested using Kruskal-Wallis and χ^2^ tests for continuous and categorical variables, respectively.

Multivariable regression analyses were used to investigate the relationship between infarction territories (any perforator, insular, cortical involvement) and primary (24-hour NIHSS) and secondary (90-day mRS) outcomes, adjusting for prognostic factors. The analyses were conducted separately for the 24-hour NIHSS and 90-day mRS. For the NIHSS, linear regression was used with baseline NIHSS adjustment for an accurate estimate of effect size. Conversely, the 90-day mRS was analyzed using ordinal logistic regression to appropriately handle its ordered categories and to provide more accurate estimates of the effect size across the full range of outcomes. The 24-hour NIHSS reflected the effect size on the acute neurological status, and the 90-day mRS reflected the effect size on the functional outcome at follow-up.

The following prognostic factors were identified in literature and analyzed in univariable regression analysis for each outcome measure: sex, age, previous ischemic stroke, hypertension, diabetes, smoking, antiplatelet use, Vitamin K use, direct oral anticoagulants use, whether intravenous thrombolysis was performed, Thrombolysis in Cerebral Infarction grade, time onset to reperfusion (in minutes), time start of symptoms to door (in minutes), Heidelberg Bleeding Classification, Fazekas grade, infarction locations (perforator only, insular, cortex), mRS prestroke, NIHSS full scale at baseline, and occlusion levels. Final infarction volume (in milliliters) was only available for patients included in the MR CLEAN-MED trial. Therefore, a sensitivity regression analysis was performed, including only MR CLEAN-MED patients in which infarct volume could be used as a prognostic factor.

Variables with *P*<0.20 in univariable regression analyses were included in the multivariable regression analyses. Multivariable linear regression analyses were performed using the full scale 24-hour NIHSS as a dependent variable. Multivariable ordinal logistic regression analyses were performed using 90-day mRS as a dependent variable. Patients with missing data and outliers in the main outcomes (24-hour NIHSS and 90-day mRS) were removed from further analysis. To account for multicollinearity in the multivariable regression analyses, the variance inflation factor (VIF) was calculated. Variables with a VIF of 5 or larger in the multivariable regression analyses were removed from the regression models.

Effect estimates were reported using regression coefficients (β) and common odds ratios with 95% CIs for the linear and ordinal analyses, respectively.

#### Anatomic Analysis

In patients with perforator and insular territory infarctions, analyses for specific infarct locations were performed. Percentages of infarction occurrence for each specific location in the perforator or insular territory were compared between occlusion levels (ICA, ICA-t, proximal M1, distal M1, M2 superior, M2 inferior) using the χ^2^ test. A Bonferroni correction for multiple comparisons was applied, with *P* values of <0.0125 considered statistically significant.

Percentages of infarction occurrence were used to generate heatmaps detailing the likelihood of an anatomic structure to be involved in the infarction of a specific perforator group.

#### Analysis of Clinical Symptoms

To evaluate the occurrence of specific symptoms and correlate these with infarction patterns of the various perforator groups, each clinical symptom described in the NIHSS full scale was analyzed separately. The occurrence of symptoms in the infarction groups was compared using the χ^2^ test between infarction location groups and occlusion levels. All analyses were performed using SPSS, version 29.0.1.0 (IBM)^15^ and R version 4.0.5.^16^

## Results

### Baseline Characteristics

**Figure 1.**
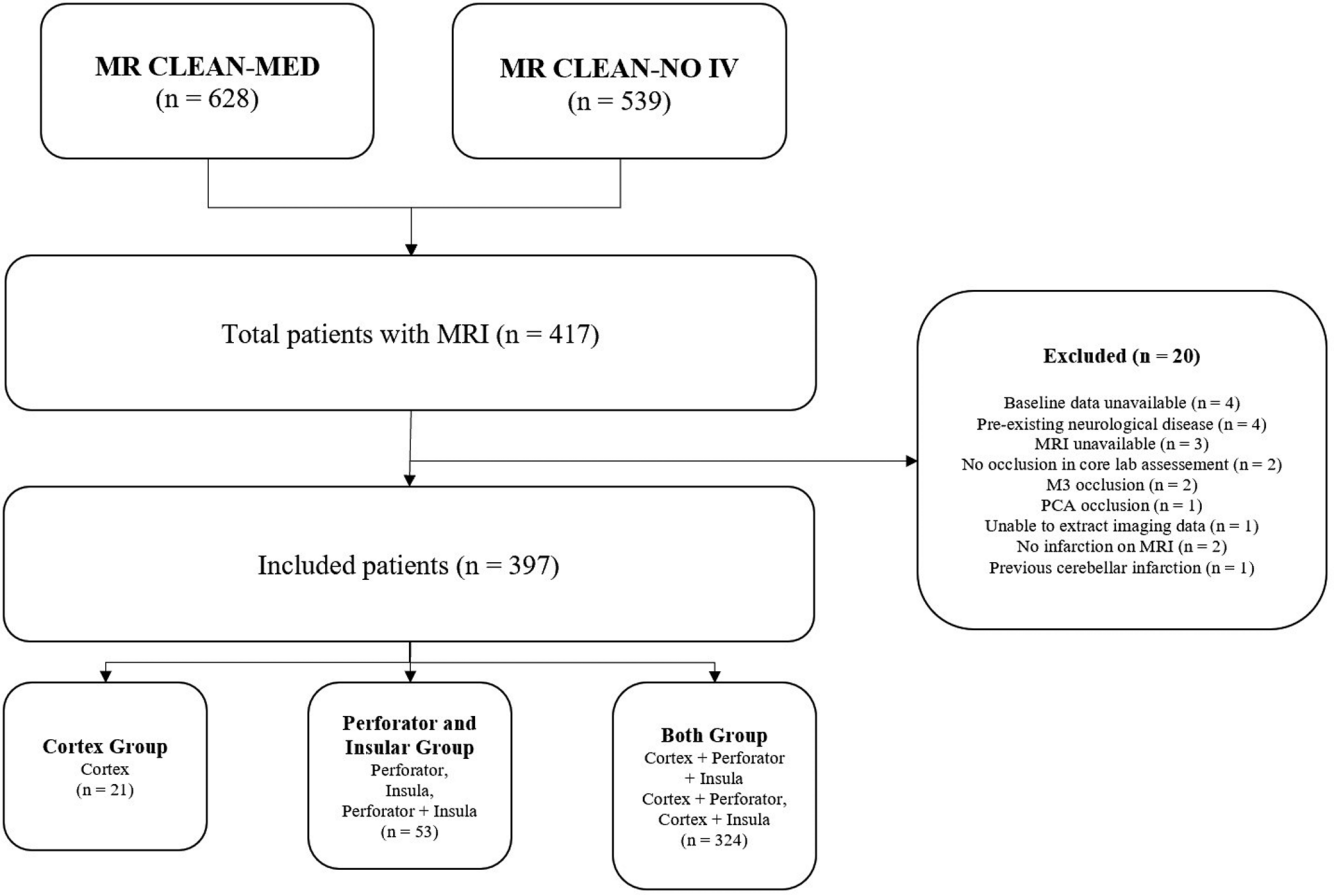
**Flowchart of patient inclusion and group division.** MR CLEAN-MED indicates Safety and Efficacy of Aspirin, Unfractionated Heparin, Both, or Neither During Endovascular Stroke Treatment; MR CLEAN-NO IV, Intravenous Treatment Followed by Intra-Arterial Treatment Versus Direct Intra-Arterial Treatment for Acute Ischemic Stroke Caused by a Proximal Intracranial Occlusion; MRI, magnetic resonance imaging; and PCA, posterior cerebral artery.

A total of 1167 patients with LVO were enrolled in the MR CLEAN-MED and MR CLEAN-NO IV trials, of which 417 met the inclusion criteria. Of these, 20 patients were further excluded due to preexisting neurological disease on MRI, no apparent occlusion on CT angiography, distal vessel or posterior circulation occlusion, unavailable baseline or imaging data, inability to extract data due to suboptimal image quality, no infarction identified on MRI, or cerebellar infarction. A total of 397 patients with LVO were included in the final analysis (Figure 1).

**Figure 2.**
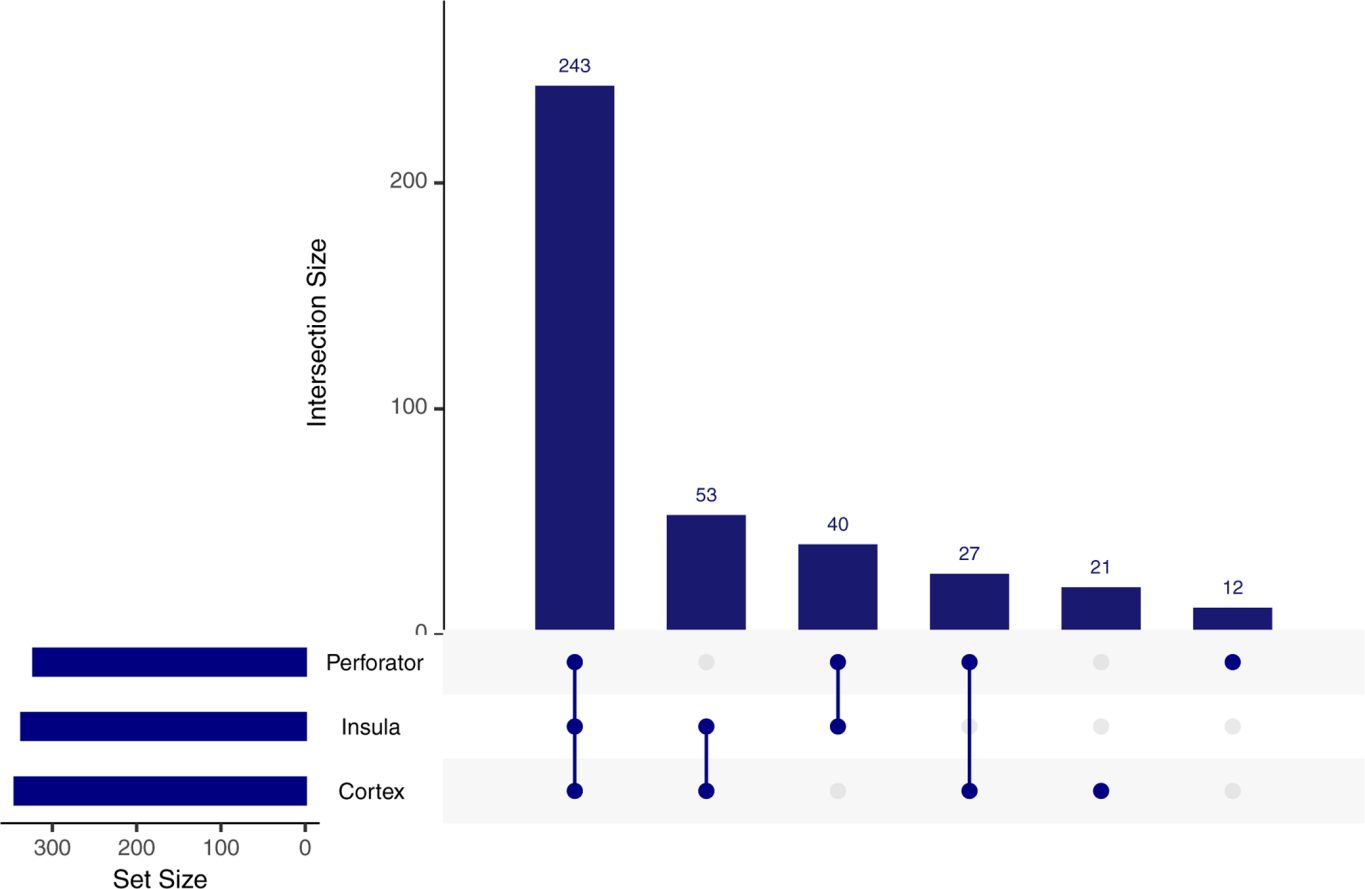
**Upset plot of the number of patients per of infarction territory.** Source: Conway et al.^17^

In the entire sample, the median age was 71 years (IQR, 62–79), and 204 (51%) were men (Table 1). Diabetes was the most common comorbidity (n=183, 46%). In total, 21 (5%) patients were included in the cortex group, 52 (13%) patients in the perforator and insular group, and 324 (82%) patients in both groups (Figures 1 and 2; Table 1). Median NIHSS at presentation were the highest in the perforator and insular group (16; IQR, 8–18) and both group (16; IQR, 10–20; *P*<0.001; Table 1). A total of 18 (4%) patients had an ICA occlusion, 83 (21%) an occlusion at the ICA-t, 144 (36%) at the proximal M1 segment, 73 (18%) at the distal M1 segment, 52 (13%) at the M2 superior, and 27 (7%) at the M2 inferior trunk, with similar distributions between the 3 groups (Table 1).

**Table 1.**
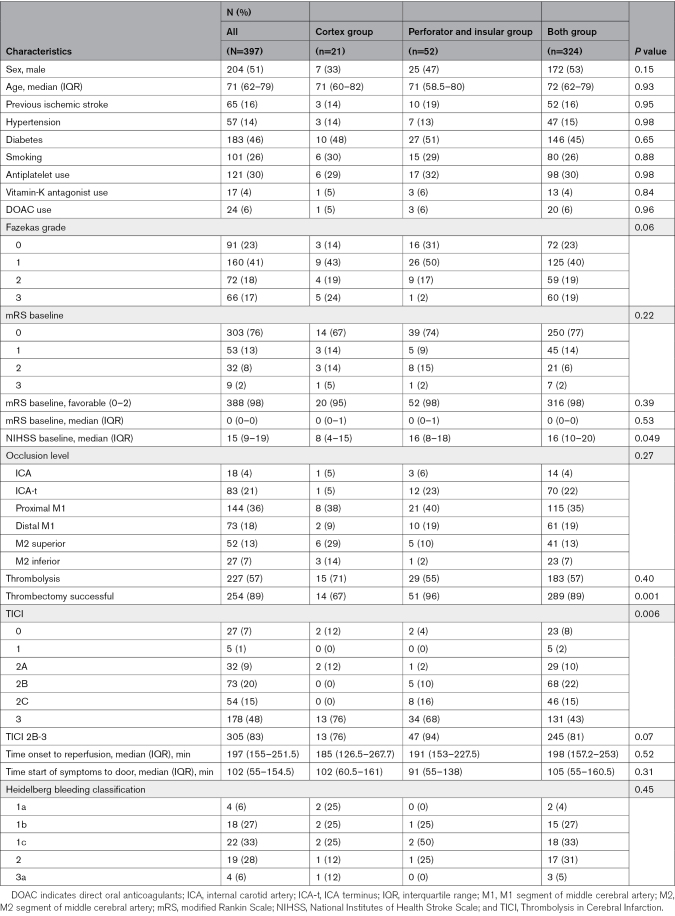
Baseline Characteristics at Presentation

The interrater reliability of the infarction location assessment was high (Cohen κ=0.91), and the agreement percentage for internal quality assurance was 88.6%, corresponding to excellent internal validity.

### Neurological Deficits and Functional Outcomes

**Figure 3.**
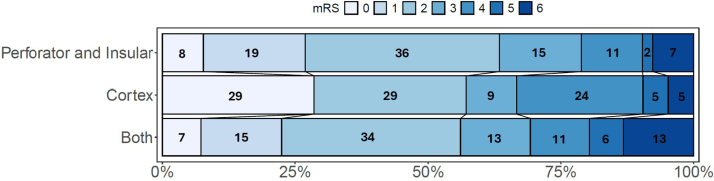
modified Rankin Scale (mRS) scores at 90-days per infarct location group.

**Figure 4.**
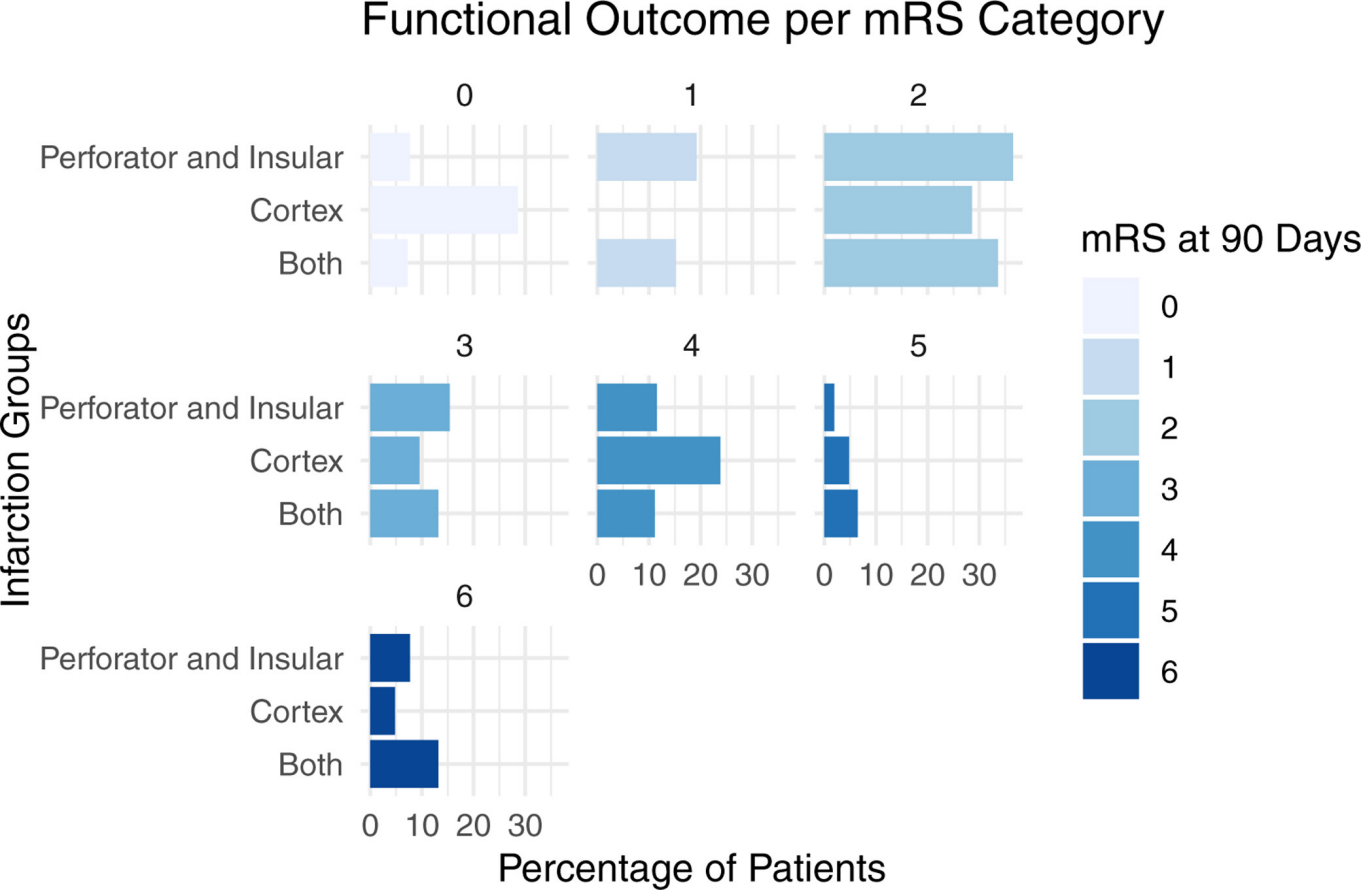
Facet plot of distribution of modified Rankin Scale (mRS) scores at 90 days per infarct location group.

Median NIHSS at 24 hours of follow-up was 5 (IQR, 1–11) for all patients, with the highest observed for both group (6; IQR, 2–12). Of all patients, 227 (57%) had a favorable functional outcome at 90-day follow-up (Figures 3 and 4; Table 2).

**Table 2.**
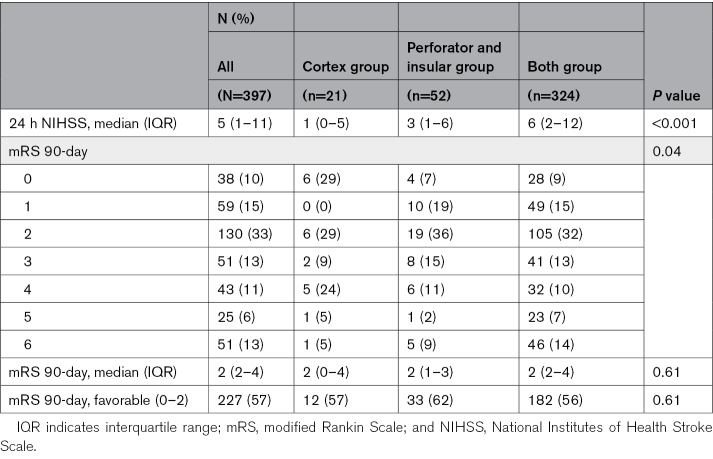
Clinical Severity and Functional Outcome at Follow-Up

Patients with any cortical infarctions had a higher full-scale NIHSS at 24 hours (β coefficient, 2.22 [95% CI, 0.35–4.08]; *P*=0.02). No significant associations were found between 24-hour NIHSS and infarctions in the perforator territory or the insular territory. In MR CLEAN-MED patients, no significant associations were found between infarction locations and 24-hour NIHSS (Table 3).

**Table 3.**
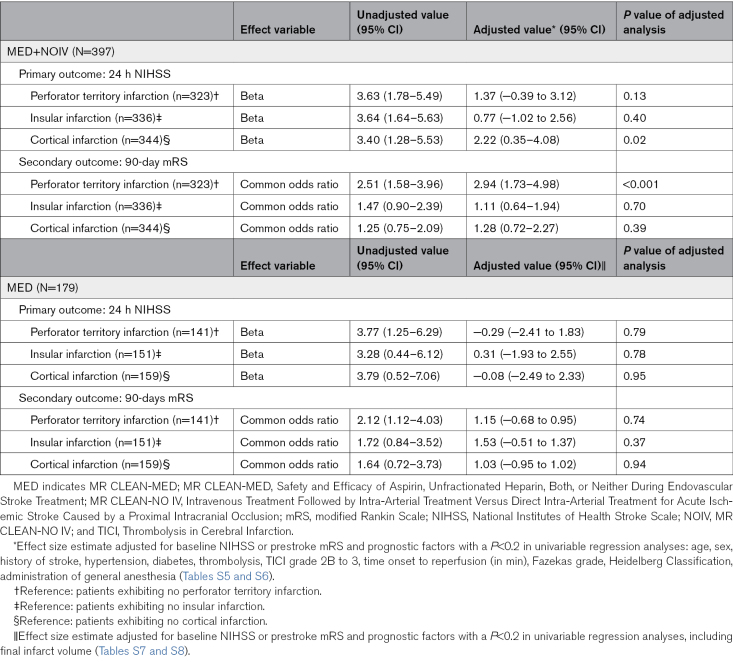
Effect of Infarct Location on Primary and Secondary Outcomes

Patients with perforator territory infarction were more likely to have a higher mRS at 90 days of follow-up (median mRS, 2 [IQR, 1–3]; common odds ratio, 2.94 [95% CI, 1.73–4.98]; *P*<0.001). No significant associations were found between 90-day mRS and infarctions in the insular or cortical region (Table 3). In MR CLEAN-MED patients, no significant associations were found between infarction locations and 90-day mRS (Table 3).

### Infarction Regions per Occlusion Level

**Figure 5.**
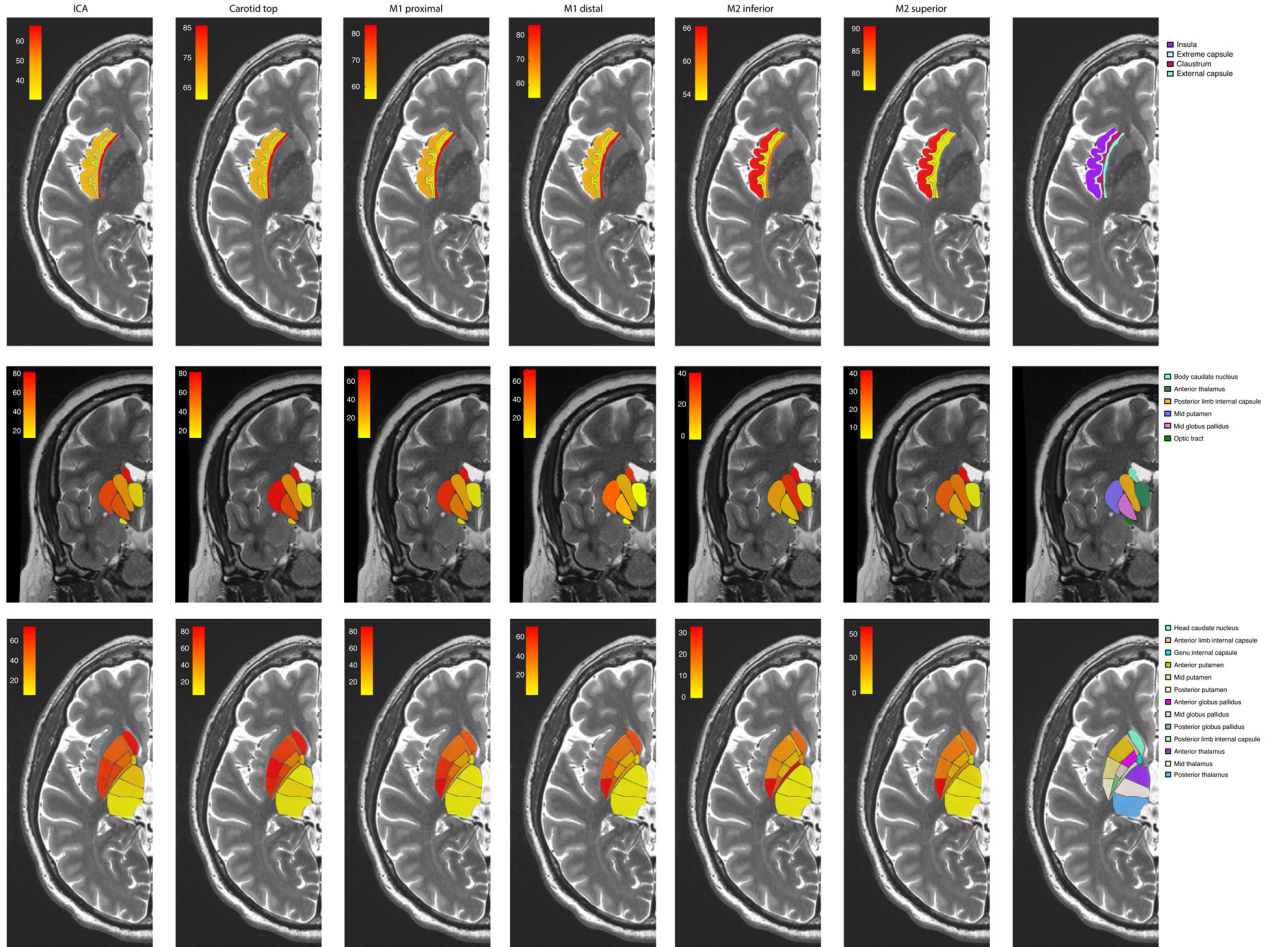
**Brain maps of infarct structures per occlusion location in patients with infarct in perforator territories.** Carotid top the internal carotid artery (ICA) terminus, M1, the M1 segment of the middle cerebral artery (MCA); M2 superior, superior trunk of the M2 segment; M2 inferior, inferior trunk of the M2 segment.

In patients with ICA-t occlusion (n=83), infarctions occurred significantly more often in the head of the caudate nucleus, anterior limb of internal capsule, genu of internal capsule, anterior one-third and mid-third of the putamen, anterior one-third and mid-third of the globus pallidus, and mid-third of the thalamus compared with patients with proximal M1 occlusions (Figure 5; Figures S1 and S2; Table S1). A mixed infarction phenotype (cortical plus perforator plus insular region infarctions) was most frequently apparent in patients with ICA-t occlusion (n=63, 76%; *P*<0.001; Table S2).

In patients with occlusions of the distal M1 segment (n=73), the head and body of the caudate nucleus, mid-third and posterior one-third of the putamen, posterior one-third of the globus pallidus, and the external capsule were affected more often than in patients with occlusions in the M2 superior segment (Figure 5; Figure S1; Tables S1 and S2).

### Clinical Symptoms

On presentation, 61% (n=196) of patients in both groups experienced sensory loss, compared with 55% (n=29) in the perforator group and 57% (n=12) in the cortex group (*P*=0.02; Table S3). Aphasia occurred in 57% (n=185) patients in both groups compared with 53% (n=28) in the perforator group and 43% (n=9) in the cortex group (*P*=0.02; Table S3). Table S4 shows the occurrence of clinical symptoms per occlusion level.

## Discussion

### Key Findings

In this study, we performed a post hoc analysis of the MR CLEAN-MED and MR CLEAN-NO IV trials, including 397 patients with LVO who underwent endovascular thrombectomy, to investigate the effect of perforator territory infarctions on the functional outcome.

We showed that there was no association between perforator territory infarctions and 24-hour NIHSS. However, patients suffering from perforator territory ischemia were more likely to have a higher 90-day mRS, adjusted for prestroke mRS and prognostic factors.

In previous studies, infarctions involving the basal ganglia, corona radiata, and insula have been linked to unfavorable functional outcomes.^18–21^ Both the MR CLEAN (Multicenter Randomized Clinical Trial of Endovascular Treatment for Acute Ischemic Stroke in the Netherlands) and HERMES trials (Highly Effective Reperfusion Evaluated in Multiple Endovascular Stroke Trials) found that infarctions in the basal ganglia are associated with higher mRS scores at follow-up.^22,23^ Additionally, several studies found that visibility of the lateral lenticulostriate arteries (LLSA) on preinterventional DSA was associated with substantial neurological improvement and a favorable outcome at follow-up in patients with occlusion of the M1 segment, highlighting the impact of the perforating arteries on functional outcome.^24,25^

Previous studies also suggest that proximal M1 occlusion is associated with a higher rate of LLSA occlusion and infarction in perforator territories.^2,26,27^ These findings are in line with our results, which indicate that both ICA-t as well as proximal M1 occlusions correlate with a larger area of ischemia in the anterior perforator territory than occlusions in the M2 segments. Our heatmap analysis revealed that in patients with proximal occlusions (ICA, ICA-T, proximal M1), higher frequencies of gaze palsy, arm and leg paresis, facial paresis, and dysarthria were observed. In contrast, distal occlusions (M2 inferior) exhibited lower rates of these symptoms. These findings align with data that proximal LVOs affect broader cortical and subcortical areas, leading to more pronounced motor and cortical deficits.^6,28^ A systematic review by Vogels et al^2^ reported that the occlusion of LLSAs caused infarction in the thalamus; however, in our current cohort, the thalamus appeared to almost never be supplied by LLSAs. This discrepancy may be caused by the relatively limited number of patients in previous studies and in the assessment of imaging data.

Despite the inevitable clinical consequences of perforator territory ischemia, there is some variation in the extent to which LVO causes ischemic damage to the perforator territory. In our cohort, ischemic damage in the perforator territories was present in 81% of LVO patients. Similar occlusions can result in major perforator territory infarctions in some patients, while in others, they may lead to no perforator territory ischemia at all. This might be caused by anatomic variation in the number and origin of perforating arteries; however, it could also imply that among perforator arteries, there is redundancy, such as collateral vascularization. In our opinion, our results conflict with the theory regarding perforating arteries as end-arteries. Their collateral circulation is quite clearly present: an occlusion in the M1-segment, where most LLSA arise, would cause infarction of the territory vascularized by these perforators, which are not only anatomically located at the base of the thrombus, but are purportedly less resistant to ischemia. Nevertheless, 14% of patients with M1 occlusions show no perforator territory ischemia, even though the pial network is affected and cortical damage is present, which strongly suggests the presence of collateral circulation.

In clinical practice, perforator territory infarction is a known risk of both endovascular and microsurgical treatment of cerebral aneurysms.^27,29–31^ Microvascular treatment of aneurysms or neuro-oncological pathologies, such as gliomas and meningiomas, highly depends on preoperative visualization and intraprocedural identification of perforating arteries to avoid significant neurological damage.^32–34^

A relevant topic in LVO research is the use of mechanical thrombectomy in medium and distal vessel occlusions. This would be applied in case of either a primary thrombus in the distal vessels or a secondary embolus after mechanical thrombectomy for an LVO. Mechanical thrombectomy for distal vessel occlusion might be more challenging to perform because distal vessels are smaller in size and more fragile.^35,36^ According to recent evidence provided by the DISTAL trial, mechanical thrombectomy for distal vessel occlusion did not result in a higher rate of favorable functional outcome than medical treatment alone.^37^

Since in our current study, perforator territory infarction showed a high rate of poor functional outcome, industry and physician-led research efforts should also be directed towards the proper visualization and further development and improvement of the current endovascular techniques to be tested in perforator revascularization.

Another possibility is the use of intra-arterial thrombolytics during thrombectomy.^38–40^ The effect may be attributed to the dissolution of small thrombi that persist after the initial endovascular procedure, resulting in improved reperfusion of the microvasculature. Thrombi that persist in the perforating arteries after thrombectomy and thereby restrict blood flow to the basal ganglia are small and with a low clot burden, making them a suitable target for thrombolytic therapy in theory. However, the currently available evidence regarding the effect of intra-arterial thrombolytics is insufficient to support their routine use in clinical practice.^41^ Therefore, it might be useful to further explore the possibilities regarding combined thrombectomy and adjuvant thrombolysis for reperfusion of occluded perforating artery trunks.

### Strengths and Limitations

In this study, we were unable to assess the exact perforator trunks that were occluded or their contralateral collateral circulation, as most patients did not have 4-vessel DSAs available. On top of that, the infarctions were highly heterogeneous, and the perforator and insular groups were relatively underpowered compared with both groups in our cohort. The 95% CIs derived from the multivariable regression analyses are also fairly broad, together with an imbalance across mRS scores between the 3 groups, which can be a source of uncertainty regarding the interpretation of results. Additionally, the effect of perforator territory ischemia on cognitive functions has not been measured in our study, even though ischemia in the perforator territory has been linked to cognitive impairment poststroke.^42,43^ In terms of main outcomes, our study does not provide long-term follow-up data, and the stroke severity score is only reported at 24 hours. As our study follow-up is limited to 90 days, which falls within the rehabilitative period following a stroke, future studies should extend the follow-up period to draw robust conclusions regarding the impact of perforator territory ischemia on long-term functional outcome. At last, the occlusion levels were initially detected on CT angiography in both MR CLEAN-NO IV and MR CLEAN-MED trials; however, in our study, we chose to include the occlusion levels based on DSA images. We did not account for thrombus migration and its effect on the functional outcome and anatomic distribution of the infarctions, as only 8.8% (n=35) of our patients had discrepancies between the occlusion level confirmed on CT angiography and DSA. We considered a level of 10% or lower of discrepancy to be acceptable to analyze the data based on occlusion levels on DSA.

Despite several limitations, this study on cerebrovascular perforator territories is unique, as perforator territory ischemia was anatomically mapped to delineate and define perforator stroke patterns. Furthermore, we provide credible evidence for the existence of collateral circulation of the perforator network. Our study is the largest study on the topic so far, as data on perforator territory ischemia remains scarce. Therefore, this study adds essential knowledge to our understanding of the cerebral perforating arteries and their clinical relevance.

### Conclusions

Patients with anterior circulation LVO leading to perforator territory infarctions are more likely to have an unfavorable functional outcome at 90 days. Future research should focus on better visualization of perforating arteries and understanding their functional anatomy to prevent perforator territory ischemia and improve patient outcomes.

## Article Information

### Sources of Funding

The CONTRAST consortium (The Collaboration for New Treatments of Acute Stroke) acknowledges the support from the Netherlands Cardiovascular Research Initiative, an initiative of the Dutch Heart Foundation (CVON2015-01: CONTRAST), and from the Brain Foundation of the Netherlands (HA2015.01.06). The collaboration project is additionally financed by the Ministry of Economic Affairs by means of the Public-Private Partnerships (PPP) Allowance made available by the Top Sector Life Sciences & Health to stimulate public-private partnerships (LSHM17016). This work was funded in part through unrestricted funding by Stryker, Medtronic, and Cerenovus. The funding sources were not involved in study design, monitoring, data collection, statistical analyses, interpretation of results, or article writing.

### Disclosures

Dr van der Lugt reports funding from Medtronic, Cerenovus, the Dutch Heart Foundation, Brain Foundation the Netherlands, the Netherlands Organization for Health Research and Development, Stryker, Penumbra, Boehringer, Trombolytic Science LLC, Health Holland Top Sector Life Sciences and Health, GE Healthcare, Siemens Healthineers, and Phillips Healthcare, all paid to the institution. Dr Majoie reports funding from Stryker and Boehringer-Ingelheim and Stryker (unrestricted grant paid to institution) and is a shareholder of Nicolab (minority interest). Dr van Doormaal reports compensation from Philips, Siemens, and Stryker for consultant services. Dr Volovici reports compensation from JAMA for consultant services. The other authors report no conflicts.

### Supplemental Material

List of MR CLEAN-NO IV Collaborators

List of MR CLEAN-MED Investigators

Tables S1–S8

Figures S1–S2

## Supplementary Material
