# Supplementary material for: Effect of Perforator Territory Infarction on Functional Outcome in Patients With Large Vessel Occlusion
Source: Stroke. 2025 Sep 19;56(12):3382–93. doi: 10.1161/STROKEAHA.125.051745 (PMC12643565; doi:10.1161/STROKEAHA.125.051745)
Supplement: Supplementary file 2 [file str-56-3382-s002.pdf]

# Supplemental Material

**List of MR CLEAN-NO IV collaborators**

Principal investigators

Yvo Roos (MD, PhD),1 Charles Majoie (MD, PhD)1

Study coordinators

Kilian Treurniet (MD),1 Jonathan Coutinho (MD, PhD),1 Bart Emmer (MD, PhD),1 Natalie LeCouffe (MD),1 Manon Kappelhof (MD),1 Leon Rinkel (MD),1 Agnetha Bruggeman (MD),1

Local principal investigators

Bob Roozenbeek (MD, PhD),2 Adriaan van Es (MD, PhD),2 Inger de Ridder (MD, PhD),4 Wim van Zwam (MD, PhD),4 Bart van der Worp (MD, PhD),5 Rob Lo (MD, PhD),5 Koos Keizer (MD, PhD),6 Rob Gons (MD),6 Lonneke Yo (MD, PhD),6 Jelis Boiten (MD, PhD),7 Ido van den Wijngaard (MD, PhD),7 Geert Lycklama à Nijeholt (MD, PhD),7 Jeanette Hofmeijer (MD, PhD),8 Jasper Martens (MD),8 Wouter Schonewille (MD, PhD),9 Jan Albert Vos, (MD, PhD),9 Anil Tuladhar (MD, PhD),10 Floris Schreuder (MD, PhD),10 Jeroen Boogaarts (MD, PhD)10, Sjoerd Jenniskens (MD),10 Karlijn de Laat (MD, PhD),11 Lukas van Dijk (MD, PhD),11 Heleen den Hertog (MD, PhD),12 Boudewijn van Hasselt (MD),12 Paul Brouwers (MD, PhD),13 Emiel Sturm (MD),13 Tomas Bulut (MD),13 Michel Remmers (MD),14 Anouk van Norden (MD),14 Thijs de Jong (MD),14 Anouk Rozeman (MD),15 Otto Elgersma (MD, PhD),15 Maarten Uyttenboogaart (MD, PhD),16 Reinoud Bokkers (MD, PhD),16 Julia van Tuijl (MD),17 Issam Boukrab (MD),17 Hans Kortman (MD),17 Vincent Costalat (MD, PhD),18 Caroline Arquizan (MD, PhD),18 Robin Lemmens (MD, PhD),19 Jelle Demeestere (MD, PhD),19 Philippe Desfontaines (MD, PhD),20 Denis Brisbois (MD, PhD),20 Frédéric Clarençon (MD, PhD),21 Yves Samson (MD, PhD),21

Local trial collaborators:

Executive and writing committee

Yvo Roos (MD, PhD),1 Charles Majoie (MD, PhD),1 Adriaan van Es (MD, PhD),2 Wim van Zwam (MD, PhD),4 Jelis Boiten (MD, PhD),7 Geert Lycklama à Nijeholt (MD, PhD),7 Lonneke Yo (MD, PhD),6 Koos Keizer (MD, PhD),6 Jonathan Coutinho (MD, PhD)1, Bart Emmer (MD, PhD)1, Kilian Treurniet (MD),1 Natalie LeCouffe (MD),1 Manon Kappelhof (MD),1

Data Safety Monitoring Board

Martin Brown (MD) – Chair,22 Phil White (MD, PhD)23, John Gregson (MD, PhD)24

Independent trial statistician

Daan Nieboer (MSc)2

CONTRAST clinical trial collaborators:

Research leaders

Diederik Dippel (MD, PhD),2 Charles Majoie (MD, PhD)1

Consortium coordinator:

Rick van Nuland (PhD)3

Imaging assessment committee

Charles Majoie (MD, PhD) – Chair,1 (Amsterdam Medical Center, location AMC); Aad van der Lugt (MD, PhD) – Chair,2 Wim van Zwam (MD, PhD),4 Linda Jacobi (MD, PhD),4 René van den Berg, (MD, PhD),1 Ludo Beenen (MD),1 Bart Emmer (MD, PhD),1 Adriaan van Es, (MD, PhD),2 Pieter-Jan van Doormaal (MD),2 Geert Lycklama (MD, PhD),7 Ido van den Wijngaard (MD, PhD),7 Albert Yoo (MD, PhD),25 Lonneke Yo (MD, PhD),6 Jasper Martens (MD, PhD),8 Bas Hammer (MD, PhD)11, Stefan Roosendaal (MD, PhD),2 Anton Meijer (MD, PhD),10

Menno Krietemeijer (MD)6, Reinoud Bokkers (MD, PhD)16, Anouk van der Hoorn (MD, PhD)16, Dick Gerrits (MD)13

Adverse event committee

Robert van Oostenbrugge (MD, PhD) – Chair,4 Bart Emmer (MD, PhD),2 Jonathan Coutinho (MD, PhD),1 Ben Jansen (MD, PhD)17

Outcome assessment committee

Yvo Roos (MD, PhD) – Chair,1 Sanne Manschot (MD, PhD),7 Diederik Dippel (MD, PhD),2

Henk Kerkhof (MD, PhD),15 Ido van den Wijngaard (MD, PhD),7 Jonathan Coutinho (MD, PhD),1 Peter Koudstaal (MD, PhD),1 Koos Keizer (MD, PhD)6

Data management group

Hester Lingsma (PhD),2 Diederik Dippel (MD, PhD)2, Vicky Chalos (MD),2 Olvert Berkhemer (MD, PhD),2

Imaging data management

Aad van der Lugt (MD, PhD),2 Charles Majoie (MD, PhD),1 Adriaan Versteeg,2 Lennard Wolff (MD),2 Jiahang Su (MSc)2, Manon Tolhuisen (MSc)1, Henk van Voorst (MD)1

Biomaterials and translational group

Hugo ten Cate (MD, PhD),4 Moniek de Maat (PhD)2, Samantha Donse-Donkel (MD),2 Heleen van Beusekom (PhD),2 Aladdin Taha (MD)2

Local collaborators

Vicky Chalos (MD),2 Kilian Treurniet (MD),1 Sophie van den Berg (MD),1 Natalie LeCouffe (MD),1 Rob van de Graaf (MD),2 Robert-Jan Goldhoorn (MD),4 Aladdin Taha (MD),2 Samantha Donse-Donkel (MD),2 Wouter Hinsenveld (MD),4 Anne Pirson (MD),4 Lotte Sondag (MD),10 Manon Kappelhof (MD),1 Rik Reinink (MD),5 Manon

Tolhuisen (MD),1 Josje Brouwer (MD),1 Lennard Wolff (MD),2 Sabine Collette,16 Wouter van der Steen (MD)2

Research nurses

Rita Sprengers,1 Martin Sterrenberg,2 Naziha El Ghannouti,2 Sabrina Verheesen,4 Wilma Pellikaan,9 Kitty Blauwendraat,9 Yvonne Drabbe,11 Joke de Meris,7 Michelle Simons,8 Hester Bongenaar,6 Anja van Loon,14 Eva Ponjee,12 Rieke Eilander,12 Suze Kooij,15, Marieke de Jong,16 Esther Santegoets,17 Suze Roodenburg15, Ayla van Ahee,1,5 Marinette Moynier,18 Annemie Devroye,19 Evelyn Marcis,19 Ingrid Iezzi,20 Annie David,20 Atika Talbi, 21

Study monitors

Leontien Heiligers2, Yvonne Martens2

Affiliations

1. Amsterdam Medical Center, location AMC, University of Amsterdam, Amsterdam the Netherlands;
2. Erasmus MC University Medical Center, Rotterdam, the Netherlands;
3. Lygature, Utrecht, the Netherlands;
4. Cardiovascular Research Institute Maastricht (CARIM), Maastricht University Medical Center,
5. Maastricht, The Netherlands;
6. University Medical Center Utrecht, Brain Center Rudolf Magnus, Utrecht, the Netherlands;
7. Catharina Hospital, Eindhoven, the Netherlands;
8. Haaglanden Medical Center, the Hague, the Netherlands;
9. Rijnstate Hospital, Arnhem, the Netherlands;
10. St. Antonius Hospital, Nieuwegein, the Netherlands;
11. Radboud University Medical Center, Nijmegen, the Netherlands;
12. HagaZiekenhuis, the Hague, the Netherlands;
13. Isala Klinieken, Zwolle, the Netherlands;
14. Medisch Spectrum Twente, Enschede, the Netherlands;
15. Amphia Hospital, Breda, the Netherlands;
16. Albert Schweitzer Hospital, Dordrecht, the Netherlands;
17. University Medical Center Groningen, the Netherlands;
18. Elisabeth-TweeSteden Hospital, Tilburg, the Netherlands;

19. Centre Hospitalier Universitaire de Montpellier, Montpellier, France;
20. Universitair Ziekenhuis Leuven, Leuven, Belgium;
21. Centre Hospitalier Chrétien, Liège, Belgium;
22. Pitié-Salpêtrière Hospital, Paris, France;
23. National Hospital for Neurology and Neurosurgery, London, United Kingdom;
24. Institute of Neuroscience and Newcastle University Institute for Ageing, Newcastle
25. University, Newcastle, United Kingdom;
26. London School of Hygiene & Tropical Medicine, London, United Kingdom;
27. Texas Stroke Institute, Plano, Texas, United States of America

**List of MR CLEAN-MED investigators**

Principal investigators

Diederik Dippel (MD, PhD),1 Aad van der Lugt (MD, PhD)1

Study coordinators

Wouter van der Steen (MD),1 Rob van de Graaf (MD, PhD),1 Bob Roozenbeek (MD, PhD)1

Local principal investigators

Bob Roozenbeek (MD, PhD),1 Adriaan van Es (MD, PhD),1,2 Pieter Jan van Doormaal (MD),1 Jonathan M. Coutinho (MD, PhD),3 Bart Emmer (MD, PhD),3 Inger de Ridder (MD, PhD),4 Wim van Zwam (MD, PhD),4 Bart van der Worp (MD, PhD),5 Irene van der Schaaf (MD, PhD),5 Rob Gons (MD, PhD),6 Lonneke Yo (MD),6 Jelis Boiten (MD, PhD),7 Ido van den Wijngaard (MD, PhD),2,7 Jeanette Hofmeijer (MD, PhD),8 Jasper Martens (MD),8 Wouter Schonewille (MD, PhD),9 Jan Albert Vos, (MD, PhD),9 Anil Man Tuladhar (MD, PhD),10 Sjoerd Jenniskens (MD),10 Karlijn de Laat (MD, PhD),11 Lukas van Dijk (MD, PhD),11 Heleen den Hertog (MD, PhD),12 Boudewijn van Hasselt (MD),12 Michel Remmers (MD),13 Douwe Vos,13 Anouk Rozeman (MD, PhD),14 Otto Elgersma (MD, PhD),14 Maarten Uyttenboogaart (MD, PhD),15 Reinoud Bokkers (MD, PhD),15 Julia van Tuijl (MD, PhD),16 Issam

Boukrab (MD),16 Benjamin Gory (MD, PhD),17 Arturo Consoli (MD),18 Mikaël Mazighi (MD, PhD),19 Frederic Clarencon (MD, PhD),20 Gaultier Marnat (MD),21

Executive and writing committee

Diederik Dippel (MD, PhD),1 Aad van der Lugt (MD, PhD),1 Rob van de Graaf (MD, PhD),1 Wouter van der Steen (MD),1 Bob Roozenbeek (MD, PhD),1 Adriaan van Es (MD, PhD),1 Yvo Roos (MD, PhD),3 Charles Majoie (MD, PhD),3 Robert van Oostenbrugge (MD, PhD),4 Wim van Zwam (MD, PhD),4 Julie Staals (MD, PhD),4 Sjoerd Jenniskens (MD),10 Lukas van Dijk (MD, PhD),11 Heleen den Hertog (MD, PhD),12

Local MR CLEAN-MED collaborators

Data Safety Monitoring Board

Peter Rothwell (MD, PhD) – Chair,22 Andrew Molyneux (MD, PhD),22 Joanna Moschandreas (MD, PhD)22

Independent trial statistician

Daan Nieboer (MSc)1

Advisory Board

Gregory del Zoppo (MD, PhD)23

CONTRAST clinical trial collaborators

Research leaders

Diederik Dippel (MD, PhD),1 Charles Majoie (MD, PhD)3

Consortium coordinator:

Rick van Nuland, (PhD)24

Imaging assessment committee

Charles Majoie (MD, PhD) – Chair,3 Aad van der Lugt (MD, PhD) – Chair,1 Adriaan van Es, (MD, PhD),1,2 Pieter

Jan van Doormaal (MD),1 René van den Berg, (MD, PhD),3 Ludo Beenen (MD),3 Bart Emmer (MD, PhD),3 Stefan Roosendaal (MD, PhD),3 Wim van Zwam (MD, PhD),4 Alida Annechien Postma (MD, PhD),25 Lonneke Yo (MD, PhD),6 Menno Krietemeijer (MD),6 Geert Lycklama (MD, PhD),7 Jasper Martens (MD),8 Sebastiaan Hammer (MD, PhD),10 Anton Meijer (MD, PhD),10 Reinoud Bokkers (MD, PhD),15 Anouk van der Hoorn (MD, PhD),15 Ido van den Wijngaard (MD, PhD),2,7 Albert Yoo (MD, PhD),26 Dick Gerrits (MD)27

Adverse event committee

Robert van Oostenbrugge (MD, PhD) – Chair,4 Bart Emmer (MD, PhD),3 Jonathan M. Coutinho (MD, PhD),3 Martine Truijman (MD, PhD),4 Julie Staals (MD, PHD),4 Bart van der Worp (MD, PhD),5 J. Boogaarts (MD, PhD),10 Ben Jansen (MD, PhD),16 Sanne Zinkstok (MD, PhD)28

Outcome assessment committee

Yvo Roos (MD, PhD) – Chair,3 Peter Koudstaal (MD, PhD),1 Diederik Dippel (MD, PhD),1 Jonathan M. Coutinho (MD, PhD),3 Koos Keizer (MD, PhD),5 Sanne Manschot (MD, PhD),7 Jelis Boiten (MD, PhD),7 Henk Kerkhoff (MD, PhD),14 Ido van den Wijngaard (MD, PhD)2,7

Data management group

Hester Lingsma (PhD),1 Diederik Dippel (MD, PhD),1 Vicky Chalos (MD),1 Olvert Berkhemer (MD, PhD)1,3

Imaging data management

Aad van der Lugt (MD, PhD),1 Charles Majoie (MD, PhD),3 Adriaan Versteeg,1 Lennard Wolff (MD),1 Matthijs van der Sluijs (MD),1 Henk van Voorst (MD),3 Manon Tolhuisen (MSc),3

Biomaterials and translational group

Hugo ten Cate (MD, PhD),4 Moniek de Maat (PhD),1 Samantha Donse-Donkel (MD),1 Heleen van Beusekom (PhD),1 Aladdin Taha (MD),1 Aarazo Barakzie (MD)1

Local collaborators

Vicky Chalos (MD, PhD),1 Rob van de Graaf (MD, PhD),1 Wouter van der Steen (MD),1 Aladdin Taha (MD),1 Samantha Donse-Donkel (MD),1 Lennard Wolff (MD),1 Kilian Treurniet (MD),3 Sophie van den Berg (MD),3 Natalie LeCouffe (MD),3 Manon Kappelhof (MD),3 Rik Reinink (MD),3 Manon Tolhuisen (MD),3 Leon Rinkel (MD),3 Josje Brouwer (MD),3 Agnetha Bruggeman (MD),3 Henk van Voorst (MD),3 Robert-Jan Goldhoorn (MD),4 Wouter Hinsenveld (MD),4 Anne Pirson (MD),4 Susan Olthuis (MD),4 Simone Uniken Venema (MD),5 Sjan Teeselink (MD),10 Lotte Sondag (MD),10 Sabine Collette (MD)15

Research nurses

Martin Sterrenberg,1 Naziha El Ghannouti,1 Laurine van der Steen,3 Sabrina Verheesen,4 Jeannique Vranken,4 Ayla van Ahee,5 Hester Bongenaar,6 Maylee Smallegange,6 Lida Tilet,6 Joke de Meris,7 Michelle Simons,8 Wilma Pellikaan,9 Wilma van Wijngaarden,9 Kitty Blauwendraat,9 Yvonne Drabbe,11 Michelle Sandiman-Lefeber,11 Anke Katthöfer,11 Eva Ponjee,12 Rieke Eilander,12 Anja van Loon,13 Karin Kraus,13 Suze Kooij,14 Annemarie Slotboom,14 Marieke de Jong,15 Friedus van der Minne,15 Esther Santegoets16

Study monitors

Leontien Heiligers1, Yvonne Martens,1 Naziha El Ghannouti1

Affiliations

1. Erasmus MC University Medical Center, Rotterdam, the Netherlands;
2. Leiden University Medical Center, Leiden, the Netherlands;
3. Amsterdam University Medical Centers, location AMC, Amsterdam, the Netherlands;
4. Cardiovascular Research Institute Maastricht (CARIM), Maastricht University Medical Centre, Maastricht, The Netherlands;
5. University Medical Center Utrecht, Brain Center Rudolf Magnus, Utrecht, the Netherlands;
6. Catharina Hospital, Eindhoven, the Netherlands;
7. Haaglanden Medical Centre, the Hague, the Netherlands;

8. Rijnstate Hospital, Arnhem, the Netherlands;
9. St. Antonius Hospital, Nieuwegein, the Netherlands;
10. Radboud University Medical Center, Nijmegen, the Netherlands;
11. HagaZiekenhuis, the Hague, the Netherlands;
12. Isala, Zwolle, the Netherlands;
13. Amphia Hospital, Breda, the Netherlands;
14. Albert Schweitzer Hospital, Dordrecht, the Netherlands;
15. University Medical Center Groningen, Groningen, the Netherlands;
16. Elisabeth-TweeSteden Hospital, Tilburg, the Netherlands;
17. University Hospital of Nancy, Nancy, France
18. Foch Hospital, Suresnes, France
19. Fondation Rothschild Hospital, Paris, France
20. University Hospital of Bordeaux, Bordeaux, France
21. Pitié-Salpêtrière University hospital, Paris, France
22. John Radcliffe Hospital, Oxford, United Kingdom
23. University of Washington, Seattle, Washington, United States
24. Lygature, Utrecht, the Netherlands;
25. School for Mental Health and Sciences (Mhens), Maastricht University Medical Center, Maastricht, The Netherlands
26. Texas Stroke Institute, Dallas-Fort Worth, Texas, United States of America
27. Medisch Spectrum Twente, Enschede, The Netherlands
28. TerGooi, Hilversum, The Netherlands

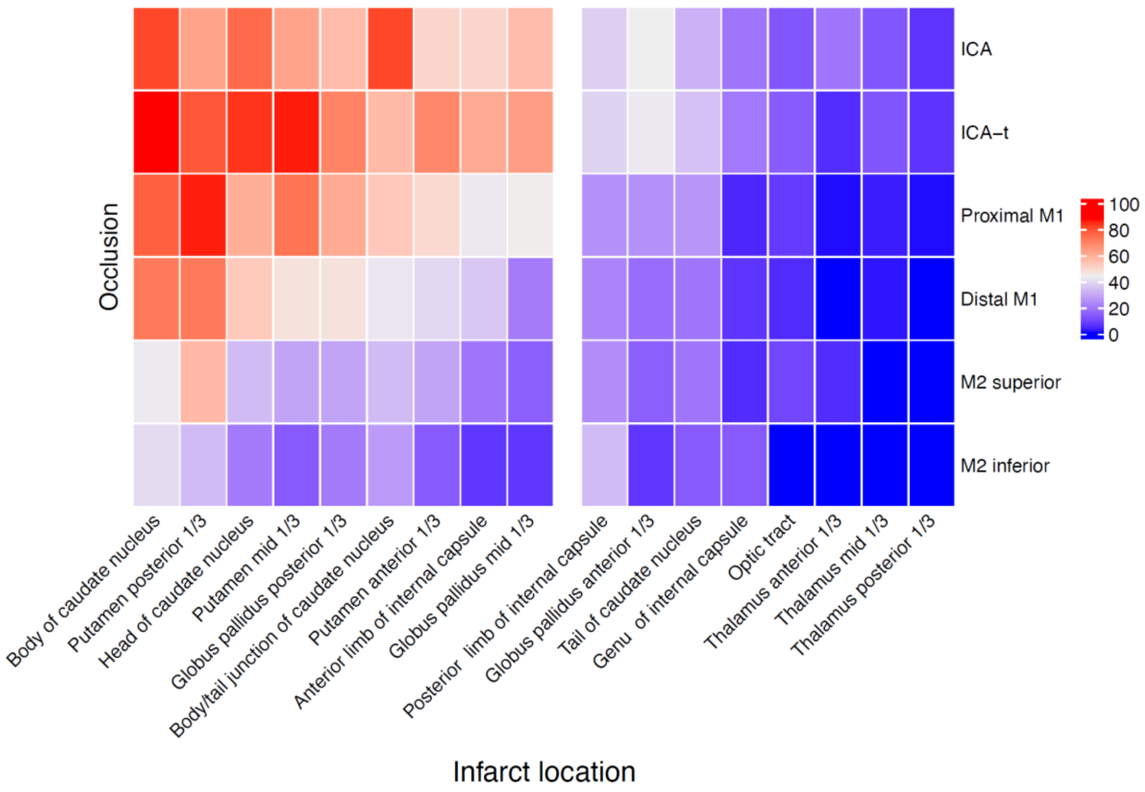


**Figure S1.** Heatmap of Infarct Structures in Perforator Territories per Occlusion Location.
ICA: Internal Carotid Artery, ICA-t: ICA terminus, M1 segment of Middel Cerebral Artery, M2: M2 segment of Middle Cerebral Artery.

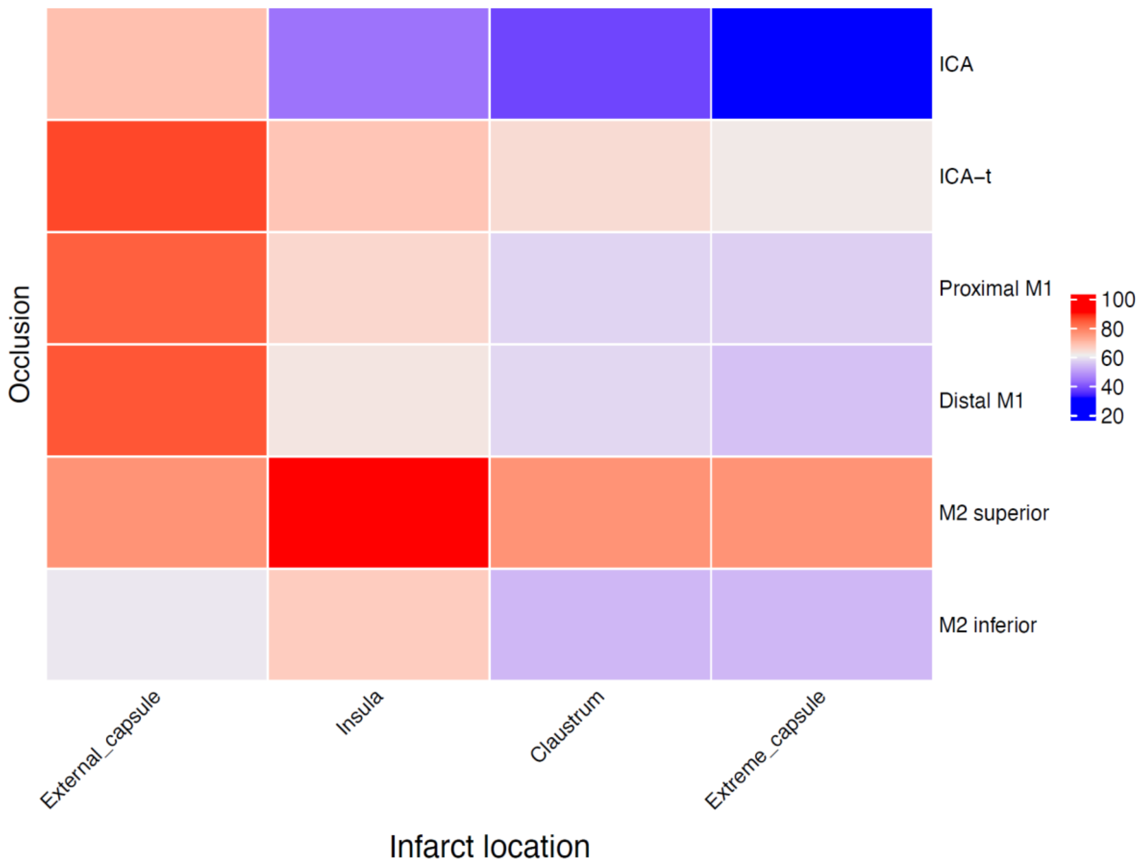


**Figure S2.** Heatmap of Infarct Structures in the Insular Region per Occlusion Location.
ICA: Internal Carotid Artery, ICA-t: ICA terminus, M1 segment of Middel Cerebral Artery, M2: M2 segment of Middle Cerebral Artery.

**Table S1.** Comparison of Infarct Location per Occlusion Location with Bonferroni Correction.

| Occlusion comparison | Infarct structures | | | | | | | | | | | | | | | | | | | | |
|---|---|---|---|---|---|---|---|---|---|---|---|---|---|---|---|---|---|---|---|---|---|
| | Perforator territory | | | | | | | | | | | | | | | | | Insular region | | | |
| | Head of caudate nucleus | Body of caudate nucleus | Body/tail junction of caudate nucleus | Tail of caudate nucleus | Anterior limb of internal capsule | Genu of internal capsule | Posterior limb of internal capsule | Optic tract | Putamen anterior 1/3 | Putamen mid 1/3 | Putamen posterior 1/3 | Globus pallidus anterior 1/3 | Globus pallidus mid 1/3 | Globus pallidus posterior 1/3 | Thalamus anterior 1/3 | Thalamus mid 1/3 | Thalamus posterior 1/3 | External capsule | Claustrum | Extreme capsule | Insula |
| ICA vs ICA-t | - | - | - | - | 0.424 | - | - | - | 0.843 | 0.183 | - | - | - | - | 0.028 | - | - | - | 0.995 | 0.569 | 0.912 |
| ICA-t vs proximal M1 | <0.001* | 0.016 | - | 0.402 | 0.003* | <0.001* | 0.060 | 0.649 | <0.001* | 0.004* | - | 0.003* | <0.001* | 0.131 | 0.606 | 0.003* | 0.048 | - | - | - | - |
| Proximal M1 vs distal M1 | - | - | - | - | - | - | - | - | - | 0.016 | - | - | 0.023 | - | - | - | - | - | - | - | - |
| Distal M1 vs M2 superior | <0.001* | <0.001* | 0.091 | - | 0.053 | - | - | - | 0.044 | 0.003* | <0.001* | - | - | 0.007* | - | - | - | <0.001* | - | - | 0.838 |
| M2 sup vs M2 inf | - | - | - | - | - | - | - | - | - | - | - | - | - | - | - | - | - | - | - | - | - |

ICA: Internal Carotid Artery, ICA-t: ICA terminus, M1: M1 segment of Middel Cerebral Artery, M2: M2 segment of Middle Cerebral Artery.

*The significance level for the comparison of occlusion locations was set at 0.0125 using a Bonferroni correction.

| **Table S2.** Frequencies of infarct regions per occlusion level*. | | | | | | | |
|---|---|---|---|---|---|---|---|
| | **No. (%)** | | | | | | |
| | **Occlusion level** | | | | | | |
| **Infarct region** | **All (n=397)** | **ICA (n=18)** | **ICA-t (n=83)** | **Proximal M1 (n=144)** | **Distal M1 (n=73)** | **M2 superior (n=52)** | **M2 inferior (n=27)** |
| Perforator territory | 12 (3) | 1 (6) | 2 (2) | 3 (2) | 3 (4) | 2 (4) | 1 (4) |
| Insular region | 1 (0.3) | 0 (0) | 0 (0) | 0 (0) | 0 (0) | 1 (2) | 0 (0) |
| Perforator + insular | 40 (10) | 2 (11) | 10 (12) | 18 (12) | 8 (11) | 2 (4) | 0 (0) |
| Cortex | 21 (5) | 1 (6) | 1 (1) | 8 (6) | 2 (3) | 6 (11) | 3 (11) |
| Cortex + insular | 53 (13) | 1 (6) | 1 (1) | 14 (10) | 7 (10) | 22 (42) | 8 (30) |
| Cortex + perforator | 27 (7) | 3 (17) | 6 (7) | 8 (6) | 5 (7) | 0 (0) | 5 (18) |
| Cortex + perforator + insular | 243 (61) | 10 (56) | 63 (76) | 93 (65) | 48 (66) | 19 (36) | 10 (37) |

ICA: Internal Carotid Artery, ICA-t: ICA terminus, M1: M1 segment of Middel Cerebral Artery, M2: M2 segment of Middle Cerebral Artery.

*P<0.001.

| Table S3. Differences Between Groups in Terms of Symptoms Upon Presentation | | | | | |
|---|---|---|---|---|---|
| | No. (%) | | | | |
| | All (n=397) | Cortex Group (n=21) | Perforator Group (n=52) | Both Group (n=324) | *P value* |
| Decreased level of consciousness | 229 (58) | 12 (57) | 32 (60) | 192 (60) | 0.29 |
| Gaze palsy | 259 (66) | 9 (43) | 32 (60) | 216 (67) | 0.08 |
| Hemianopia | 245 (62) | 12 (57) | 33 (62) | 206 (64) | 0.22 |
| Facial paresis | 359 (91) | 17 (81) | 48 (91) | 292 (91) | 0.12 |
| Arm paresis | 346 (88) | 16 (76) | 42 (79) | 285 (88) | 0.24 |
| Leg paresis | 309 (78) | 15 (71) | 36 (68) | 253 (79) | 0.74 |
| Limb ataxia | 22 (6) | 3 (14) | 1 (2) | 18 (6) | 0.11 |
| Sensory loss | 229 (58) | 12 (57) | 29 (55) | 196 (61) | 0.02 |
| Aphasia | 214 (54) | 9 (43) | 28 (53) | 185 (57) | 0.02 |
| Dysarthria | 305 (78) | 15 (71) | 40 (75) | 247 (77) | 0.41 |
| Extinction and inattention | 229 (58) | 12 (57) | 27 (51) | 190 (59) | 0.63 |

**Table S4.** Comparison Heatmap of Symptoms Upon Presentation per Occlusion Level

| **Occlusion level** | **ICA (%)** | **ICA-t (%)** | **Proximal M1 (%)** | **Distal M1 (%)** | **M2 superior (%)** | **M2 inferior (%)** | ***P value*** |
|---|---|---|---|---|---|---|---|
| Decreased level of consciousness | 72 | 65 | 57 | 60 | 46 | 52 | 0.25 |
| Gaze palsy | 72 | 74 | 74 | 59 | 44 | 48 | <0.001 |
| Hemianopia | 67 | 63 | 68 | 60 | 46 | 56 | 0.11 |
| Facial paresis | 89 | 93 | 96 | 88 | 85 | 78 | 0.006 |
| Arm Paresis | 78 | 91 | 94 | 86 | 85 | 59 | <0.001 |
| Leg Paresis | 72 | 87 | 85 | 82 | 61 | 44 | <0.001 |
| Limb ataxia | 17 | 4 | 6 | 4 | 6 | 4 | 0.42 |
| Sensory loss | 61 | 51 | 68 | 51 | 52 | 52 | 0.05 |
| Aphasia | 61 | 56 | 50 | 57 | 54 | 56 | 0.90 |
| Dysarthria | 72 | 79 | 89 | 79 | 60 | 63 | <0.001 |
| Extinction and inattention | 61 | 61 | 66 | 48 | 52 | 44 | 0.08 |

ICA: Internal Carotid Artery, ICA-t: Internal Carotid Artery Terminus, M1: M1 segment of Middel Cerebral Artery, M2: M2 segment of Middle Cerebral Artery.

Red, more often; blue, less often.

| **Table S5.** Multivariable linear regression analysis of MR CLEAN NOIV and MED Patients (N=397). | | | | |
|---|---|---|---|---|
| Dependent Variable: NIHSS 24h | | | | |
| | | | 95% Confidence Interval | |
| Parameter | Beta | P value | Lower Bound | Upper Bound |
| Perforator ischemia | 1.368 | 0.126 | -0.386 | 3.123 |
| Insular ischemia | 0.771 | 0.397 | -1.016 | 2.558 |
| Cortical ischemia | 2.217 | 0.020 | 0.349 | 4.084 |
| Age | 0.014 | 0.619 | -0.041 | 0.068 |
| Hypertension | 1.515 | 0.109 | -0.338 | 3.368 |
| Diabetes Mellitus | 1.040 | 0.125 | -0.289 | 2.369 |
| Thrombolysis | -0.410 | 0.518 | -1.657 | 0.837 |
| TICI 2B-3 | -5.358 | <0.001 | -7.027 | -3.689 |
| Time onset to reperfusion (min) | 0.023 | <0.001 | 0.016 | 0.030 |
| Fazekas grade 0 | -0.732 | 0.468 | -2.717 | 1.252 |
| Fazekas grade 1 | -1.288 | 0.139 | -2.996 | 0.420 |
| Fazekas grade 3 | -0.409 | 0.704 | -2.523 | 1.705 |
| Heidelberg classification 1c | 3.162 | 0.019 | 0.528 | 5.795 |
| Heidelberg classification 2 | 7.436 | <0.001 | 4.549 | 10.322 |
| Heidelberg classification 3a | 0.505 | 0.865 | -5.344 | 6.354 |
| NIHSS baseline | 0.417 | <0.001 | 0.306 | 0.527 |
| Occlusion level - M2 inferior | -0.470 | 0.713 | -2.976 | 2.037 |
| General anesthesia | 2.216 | 0.032 | 0.186 | 4.245 |

| **Table S6.** Multivariable ordinal logistic regression analysis of MR CLEAN NOIV and MED Patients (N=397), 90-days mRS as dependent variable. | | | | |
|---|---|---|---|---|
| | | | 95% Confidence Interval | |
| | Common OR | P value | Lower Bound | Upper Bound |
| Perforator ischemia | 2.936 | <0.001 | 1.730 | 4.983 |
| Insular ischemia | 1.115 | 0.699 | 0.641 | 1.937 |
| Cortical ischemia | 1.283 | 0.392 | 0.725 | 2.266 |
| Sex | 0.666 | 0.037 | 0.455 | 0.976 |
| Hypertension | 2.375 | 0.004 | 1.322 | 4.263 |
| Diabetes Mellitus | 1.332 | 0.169 | 0.886 | 2.004 |
| Age | 1.003 | 0.681 | 0.987 | 1.019 |
| Previous ischemic stroke | 1.592 | 0.082 | 0.943 | 2.691 |
| Thrombolysis | 0.739 | 0.142 | 0.495 | 1.106 |
| Vit K antagonist use | 1.256 | 0.640 | 0.483 | 3.264 |
| DOAC | 1.442 | 0.431 | 0.580 | 3.589 |
| TICI 2B to 3 | 0.329 | <0.001 | 0.194 | 0.559 |
| Time onset to reperfusion (min) | 1.001 | 0.518 | 0.999 | 1.003 |
| Fazekas grade 1 | 0.563 | 0.007 | 0.371 | 0.853 |
| Fazekas grade 3 | 1.519 | 0.171 | 0.834 | 2.768 |
| Heidelberg classification 1c | 2.368 | 0.043 | 1.029 | 5.447 |
| Heidelberg classification 2 | 10.538 | <0.001 | 3.927 | 28.276 |
| Heidelberg classification 3a | 5.286 | 0.126 | 0.627 | 44.612 |
| Prestroke mRS 2 | 3.834 | <0.001 | 1.788 | 8.224 |
| Occlusion level - ICA | 0.235 | 0.002 | 0.095 | 0.583 |
| Occlusion level - ICA-t | 1.344 | 0.214 | 0.843 | 2.147 |

**Table S7.** Multivariable linear regression analysis of MR CLEAN MED Patients (N=197).

Dependent Variable: NIHSS 24h

| Parameter | Beta | P value | 95% Confidence Interval Lower Bound | 95% Confidence Interval Upper Bound |
|---|---|---|---|---|
| Perforator ischemia | -0.291 | 0.786 | -2.407 | 1.826 |
| Insular ischemia | 0.311 | 0.784 | -1.927 | 2.548 |
| Cortical ischemia | -0.079 | 0.949 | -2.487 | 2.330 |
| Sex | -1.489 | 0.064 | -3.068 | 0.090 |
| Age | 0.086 | 0.009 | 0.022 | 0.150 |
| Hypertension | 1.923 | 0.103 | -0.398 | 4.244 |
| Diabetes Mellitus | -0.957 | 0.310 | -2.817 | 0.902 |
| Thrombolysis | -1.711 | 0.063 | -3.518 | 0.095 |
| TICI 2B-3 | -1.920 | 0.079 | -4.063 | 0.223 |
| Time onset to reperfusion (min) | 0.007 | 0.162 | -0.003 | 0.017 |
| Fazekas grade 1 | 0.038 | 0.965 | -1.709 | 1.786 |
| Fazekas grade 2 | 0.211 | 0.847 | -1.951 | 2.374 |
| Heidelberg classification 1c | 1.991 | 0.179 | -0.925 | 4.907 |
| Heidelberg classification 2 | 2.822 | 0.154 | -1.070 | 6.713 |
| NIHSS baseline | 0.351 | <0.001 | 0.210 | 0.492 |
| Occlusion level - ICA-t | 1.286 | 0.224 | -0.797 | 3.370 |
| Occlusion level - M2 inferior | -1.465 | 0.328 | -4.420 | 1.489 |
| Infarct volume (mL) | 0.053 | <0.001 | 0.039 | 0.067 |

**Table S8.** Multivariable ordinal logistic regression analysis of MR CLEAN MED Patients (N=197), 90-days mRS as dependent variable.

| | Common OR | P value | 95% Confidence Interval | |
|---|---|---|---|---|
| | | | Lower Bound | Upper Bound |
| Perforator ischemia | 1.147 | 0.741 | 0.509 | 2.588 |
| Insular ischemia | 1.530 | 0.375 | 0.598 | 3.920 |
| Cortical ischemia | 1.035 | 0.945 | 0.387 | 1.473 |
| Sex | 0.484 | 0.029 | 0.252 | 0.930 |
| Age | 0.999 | 0.921 | 0.971 | 2.641 |
| Previous ischemic stroke | 2.291 | 0.075 | 0.919 | 5.703 |
| Hypertension | 2.998 | 0.030 | 1.115 | 8.053 |
| Diabetes Mellitus | 0.883 | 0.739 | 0.426 | 1.833 |
| Thrombolysis | 0.928 | 0.878 | 0.357 | 2.416 |
| Vit K antagonist use | 0.758 | 0.680 | 0.204 | 2.826 |
| DOAC | 1.655 | 0.471 | 0.421 | 6.501 |
| Fazekas grade 1 | 0.654 | 0.264 | 0.310 | 1.379 |
| Fazekas grade 3 | 1.502 | 0.373 | 0.613 | 3.680 |
| Heidelberg classification 1c | 1.719 | 0.368 | 0.529 | 5.585 |
| Heidelberg classification 2 | 2.428 | 0.352 | 0.375 | 15.737 |
| Prestroke mRS 0 | 0.807 | 0.693 | 0.279 | 2.335 |
| Prestroke mRS 2 | 4.554 | 0.044 | 1.039 | 19.985 |
| Prestroke mRS 3 | 3.271 | 0.205 | 0.524 | 20.409 |
| Occlusion level - ICA | 0.295 | 0.038 | 0.093 | 0.936 |
| Occlusion level - ICA-t | 2.399 | 0.059 | 0.967 | 5.954 |
| Infarct volume (mL) | 1.015 | <0.001 | 1.009 | 1.021 |
